# Human Leukocyte Antigen Class I and II Alleles and Cervical Adenocarcinoma

**DOI:** 10.3389/fonc.2014.00119

**Published:** 2014-06-19

**Authors:** Mahboobeh Safaeian, Lisa G. Johnson, Kai Yu, Sophia S. Wang, Patti E. Gravitt, John A. Hansen, Mary Carrington, Stephen M. Schwartz, Xiaojiang Gao, Allan Hildesheim, Margaret M. Madeleine

**Affiliations:** ^1^Division of Cancer Epidemiology and Genetics, National Cancer Institute, Bethesda, MD, USA; ^2^Public Health Sciences Division, Fred Hutchinson Cancer Research Center, Seattle, WA, USA; ^3^Division of Cancer Etiology, Department of Population Sciences, Beckman Research Institute and the City of Hope, Duarte, CA, USA; ^4^School of Medicine, The University of New Mexico, Albuquerque, NM, USA; ^5^Cancer and Inflammation Program, Laboratory of Experimental Immunology, Leidos Biomedical Research, Inc., Frederick National Laboratory for Cancer Research, Frederick, MD, USA; ^6^Department of Epidemiology, University of Washington, Seattle, WA, USA

**Keywords:** HLA class I, HLA class II, cervical adenocarcinoma, host genetics, HPV

## Abstract

**Background:** Associations between human leukocyte antigens (HLA) alleles and cervical cancer are largely representative of squamous cell carcinoma (SCC), the major histologic subtype. We evaluated the association between HLA class I (A, B, and C) and class II (DRB1 and DQB1) loci and risk of cervical adenocarcinoma (ADC), a less common but aggressive histologic subtype.

**Methods:** We pooled data from the Eastern and Western US Cervical Cancer studies, and evaluated the association between individual alleles and allele combinations and ADC (*n* = 630 ADC; *n* = 775 controls). Risk estimates were calculated for 11 *a priori* (based on known associations with cervical cancer regardless of histologic type) and 38 non *a priori* common alleles, as odds ratios (OR) and 95% confidence intervals (CI), adjusted for age and study. In exploratory analysis, we compared the risk associations between subgroups with HPV16 or HPV18 DNA in ADC tumor tissues in the Western US study cases and controls.

**Results:** Three of the *a priori* alleles were significantly associated with decreased risk of ADC [DRB1*13:01 (OR = 0.61; 95% CI: 0.41–0.93), DRB1*13:02 (OR = 0.49; 95% CI: 0.31–0.77), and DQB1*06:03 (OR = 0.64; 95% CI: 0.42–0.95)]; one was associated with increased risk [B*07:02 (OR = 1.39; 95% CI: 1.07–1.79)]. Among alleles not previously reported, DQB1*06:04 (OR = 0.46; 95% CI: 0.27–0.78) was associated with decreased risk of ADC and remained significant after correction for multiple comparisons, and C*07:02 (OR = 1.41; 95% CI: 1.09–1.81) was associated with increased risk. We did not observe a difference by histologic subtype. ADC was most strongly associated with increased risk with B*07:02/C*07:02 alleles (OR = 1.33; 95% CI: 1.01–1.76) and decreased risk with DRB1*13:02/DQB1*06:04 (OR = 0.41; 95% CI: 0.21–0.80).

**Conclusion:** Results suggest that HLA allele associations with cervical ADC are similar to those for cervical SCC. An intriguing finding was the difference in risk associated with several alleles restricted to HPV16 or HPV18-related tumors, consistent with the hypothesis that HLA recognition is HPV type-specific.

## Introduction

Cervical cancer is the second leading cause of cancer in women world-wide, and infection with oncogenic human papillomavirus (HPV) types is required for the development of these malignancies. While HPV infections with oncogenic types are common in women, only a small subset of infected individuals develop cervical cancer for reasons not yet fully understood (1). There are two major histologic subtypes of cervical cancer: cervical adenocarcinomas (ADC), which constitute about 5–20% of all cases and squamous cell carcinomas (SCC), which constitute the majority of cases (2). Recent data from large pooled studies have shown some risk factors, such as increasing number of lifetime of partners and oral contraceptive use – are shared between the two histologic subtypes (3, 4). Cigarette smoking is one risk factor which has been consistently associated with an increased risk of SCC but not ADC (5). While carcinogenic HPV infections are the central etiologic agents for both ADC and SCC, there is divergence in HPV type detection in the two histologic subtypes, with HPV18 significantly more common in ADC than in SCC (6).

In addition to known HPV cofactors, it is hypothesized that host genetic factors play a significant role in susceptibility to cervical precancer and cancer. Studies in Scandinavian countries, with well-established population registries, provide evidence for familial aggregation in cervical cancer incidence (and that of its immediate precursor, CIN3) (7–11). Those studies reported that cervical cancer risk associations are strongest for full-relatives, intermediate for half-siblings, and lowest for non-biological relatives.

Further evidence for genetic factors in cervical cancer is provided by studies that show associations with specific human leukocyte antigens (HLA) alleles (12–20). The highly polymorphic HLA molecules play a major role in immune response by presenting foreign antigens, including HPV peptides, to T lymphocytes, thus promoting immune recognition and subsequent clearance of infected cells (16). It is thought that HLA molecules that bind HPV-specific antigens may be associated with protection from cervical cancer, while HLA molecules that fail to recognize and present HPV-specific antigens may be associated with increased cervical cancer risk. Notably, a consistent inverse association has been shown for the HLA DRB1*13:01-DQB1*06:03 haplotype, while increased risks have been shown between HLA-B*07, DQB1*03:02, and for DQB1*03:01 and cervical cancer (16, 20).

As SCC constitutes the major histologic type of cervical cancers, it remains unclear if HLA associations are different between SCC and ADC. Two recent genome-wide association studies of cervical cancer have identified strong associations between cervical cancer and single nucleotide polymorphisms in 6p21.3, the same region of the HLA loci (13, 14), however, one did not include any ADC cases (14), and the other combined across SCC and ADC, with no information on how many of each histologic type were analyzed (13).

Our objective was to investigate HLA class I and II associations with ADC using pooled data from two US case–control studies of cervical cancer.

## Materials and Methods

### Study population

We pooled data from two US case–control studies of cervical cancer, one conducted by the Fred Hutchinson Cancer Research Center (FHCRC): referred to here as the “Western US cervical cancer study” (20); and the other conducted by the US National Cancer Institute: the “Eastern US cervical cancer study” (21). Briefly, the Western US cervical cancer study enrolled women diagnosed with *in situ* or invasive cervical ADC or invasive cervical SCC between January 1986 and June 1998 or between January 2000 and December 2004. Case women were identified from the FHCRC-based cancer surveillance system (CSS), a surveillance, epidemiology, and end results (SEER) registry (22). *In situ* cases lived in the 13-county area of Western Washington State covered by the CSS registry at the time of diagnosis, and invasive cases were limited to those residing in the 3-county area that surrounded Seattle. Controls were identified through random digit dialing (RDD), and frequency matched to the cases by age (5 years strata) and area of residence. In addition, control women with a hysterectomy were excluded (*n* = 139). After restricting to women for whom there was testing for HLA class I and II alleles, there were a total of 468 women with ADC (ICD-O morphology codes 8140–8561), 481 women with SCC (ICD-O morphology codes 8010–8077), and 513 controls in the Western US study (Figure S1 in Supplementary Material).

The Eastern US cervical cancer study was a multi-center case–control study, conducted between July 1994 and March 1996. Cases were incident invasive primary cervical cancer identified from six medical centers (George Washington University Medical Center, Washington, DC; Georgetown University Medical Center, Washington, DC; Graduate Hospital, Philadelphia, PA; Hershey Medical Center, Hershey, PA; University of Maryland Hospital, Baltimore, MD; and Yale/New Haven Hospital, New Haven, CT) in the Eastern US. Population controls were females (women with hysterectomies were not eligible to be controls) identified through RDD, matched to the cases (2:1) on age (5 year strata), clinic, race/ethnicity, and telephone exchange. There were a total of 162 women with ADC (ICD-O morphology codes 8140–8561), 179 women with SCC (ICD-O morphology codes 8010–8077), and 262 controls in the Eastern US study (Figure S1 in Supplementary Material).

### HLA testing

Human leukocyte antigens class I and II loci were molecularly typed with DNA extracted from buffy coat collected from each participant as previously described (19, 20) using polymerase chain reaction (PCR) and single-stranded oligonucleotide probe-based protocols developed by the 13th International Histocompatibility Workshop.

### HPV testing

In the Western US cervical cancer study, HPV typing was performed on tumor tissue blocks from cases using PCR–RFLP as described in detail elsewhere (20); HPV DNA testing was not available for controls in this study. MY09/11 L1 consensus primers and HPV16 and HPV18 E6 type-specific primers were used with co-amplification of the β-globin as a control for sample integrity.

In the Eastern US cervical cancer study, HPV typing was performed on cervicovaginal cells (21) either self-collected using a Dacron swab or clinician collected at the time of a pelvic exam from cases and controls. Specimens were tested for presence of 27 HPV types using MY09/MY11 L1 consensus primer-based reverse line blot PCR methods (23).

### Statistical methods

To increase homogeneity, we restricted the analytic sample to individuals of Caucasian ancestry ( >85% of the study populations). To address our primary objective of investigating HLA associations with cervical ADC, the final analytic sample comprised 630 ADC cases and 775 controls. Between the two studies, we identified 166 HLA class I and II alleles that were tested in both studies. Only alleles with >5% prevalence in controls were included in the main analyses (*n* = 49 alleles). Before the data were analyzed, we searched PubMed and identified 11 “*a priori*” alleles that were found to be significantly associated with cervical cancer in at least two previously published peer-reviewed reports. Search terms included HLA, cervical cancer, cervical SCC, cervical ADC, and included only studies that examined high-resolution HLA associations with SCC or ADC in English. Reference lists of related articles and recent review articles were also screened for additional citations (Table S1 in Supplementary Material). We reasoned that the HLA associations observed to date for SCC (or without regard to histology) would also be observed for ADC.

We evaluated the association between the “*a priori*” set and the remaining 38 alleles and ADC. Odds ratios (OR) and 95% confidence intervals (95% CI) for the association between individual alleles (modeled as two-level alleles; at least one allele present vs. both alleles absent) and ADC risk were estimated using unconditional logistic regression and adjusting for study, and age at diagnosis. For the 11 *a priori* set of alleles, we performed analyses without adjustment for multiple comparisons. To adjust for multiple comparison for the remaining 38 alleles, we adopted a parametric bootstrap procedure to estimate the distribution of the minimum *p*-value among the 38 tests, under the global null hypothesis (24). A parametric bootstrap procedure was used to generate null datasets under the null model, which is the reduced logistic regression model without the effect from any HLA allele. We also used the single-step method to obtain the adjusted *p*-value for each of 38 testing alleles based on the estimated distribution of the minimum *p*-value among the 38 tests.

We further investigated the association of our significant ADC findings among SCC cases (*n* = 512) and controls available from the pooled dataset. We reasoned that this comparison would allow us to determine which, if any, allele associations are unique to ADC. This investigation was carried out using polytomous logit regression models to test the hypothesis that, for any particular allele, the strength of the association differed between ADC and SCC (test of homogeneity).

Give the differences in HPV cofactors noted between ADC and SCC, in exploratory analysis, we examined whether HLA associations differed by HPV type detected in tumor tissue. Analyses were restricted to either HPV16-related or HPV18-related ADC, with each subset of the case group compared to all controls. To ensure specificity, we restricted this analysis to the data available from the Western US study, as in the Eastern US study for a large fraction of cases, samples were collected after cervical cancer treatment and thus HPV results do not reflect HPV type present at the time of diagnosis, whereas cancer tissues were typed for presence or absence of HPV in the Western US study. We excluded 26 ADC cases with both HPV16 and HPV18 detected in the tumor tissue, and used polytomous logit regression models to test the hypothesis that, for any particular allele, the strength of the association differed between HPV16 and HPV18 ADC (test of homogeneity). Because of the lack of the control participant’s HPV status at the time/age the case was infected (presumably soon after beginning sexual activity, not at the time of cancer diagnosis) which is the most ideal time for HPV exposure assessment (25), and because HPV infections are very common, we believe that comparison with all controls in the Western study, regardless of their current HPV status, is appropriate.

Lastly, the joint effects of allele combinations were examined for each of the statistically significant alleles. We constructed variables to describe unphased combinations of significant alleles across the loci. To examine the independent and combined effects of these alleles on ADC risk, we constructed a categorical variable with mutually exclusive groups of single and multiple alleles compared to a reference group. The reference group comprised participants that did not carry any of the alleles that were present in the combination of interest. Two-sided statistical tests were performed at α = 0.05 level. SAS version 9.1.3 was used for all analyses.

## Results

The Western US study consisted of 468 cervical ADC cases (*n* = 311 *in situ*, *n* = 157 invasive) and 513 controls. The Eastern US study consisted of 162 (*n* = 46 *in situ*, *n* = 116 invasive) cervical ADC cases, and 262 controls (Figure S1 in Supplementary Material).

Selected characteristics for cases and controls are presented in Table S2 in Supplementary Material. Briefly, participants in the Western US study were similar in age to the participants from the Eastern US study (median age 39 vs. 38 years (interquartile range 32–48 vs. 30–45 years, respectively).

Table S3 in Supplementary Material presents HLA alleles with frequencies of >5% among controls for alleles that were typed in both studies. Out of the 166 alleles in both studies, 49 had frequencies >5% among the controls. The allele frequencies were largely similar across the two studies, with only seven of the allele frequencies statistically significantly different between the two study populations. We therefore combined the cohorts in subsequent analyses.

Table 1A shows the main effects for the 11 pre-defined HLA alleles. Three HLA class II alleles were associated with a lower risk of ADC (OR_DRB1*13:01_ = 0.61, 95% CI: 0.41–0.93; OR_DRB1*13:02_ = 0.49, 95% CI: 0.31–0.77; and OR_DQB1*06:03_ = 0.64, 95% CI: 0.42–0.95). One class I allele was associated with increased risk of ADC (OR_B*07:02_ = 1.39, 95% CI: 1.07–1.79).

**Table 1:** **Association between 11 *a priori* selected HLA alleles (A), and remaining 38 non *a priori* HLA alleles (B) and cervical adenocarcinoma (ADC) among Caucasians, Pooled Western and Eastern US cervical cancer studies**.

**(A)**
	ADC cases, *n* = 630	Controls, *n* = 775	Odds ratio (95% CI)	*p*-Value
**HLA-B**				
07:02	177 (28.1)	162 (20.9)	1.39 (1.07–1.79)	0.01
**DRB1**				
04:01	118 (18.73)	131 (16.9)	1.12 (0.84–1.48)	0.43
11:01	55 (8.73)	57 (7.35)	1.28 (0.86–1.89)	0.23
13:01	39 (6.19)	73 (9.42)	0.61 (0.41–0.93)	0.02
13:02	28 (4.44)	66 (8.52)	0.49 (0.31–0.77)	0.002
15:01	166 (26.35)	173 (22.32)	1.20 (0.93–1.54)	0.16
**DQB1**				
03:01	201 (31.9)	226 (29.16)	1.15 (0.91–1.46)	0.24
03:02	146 (23.17)	158 (20.39)	1.15 (0.88–1.49)	0.3
03:03	58 (9.21)	65 (8.39)	1.05 (0.72–1.53)	0.8
06:02	161 (25.56)	172 (22.19)	1.15 (0.89–1.48)	0.29
06:03	41 (6.51)	75 (9.68)	0.64 (0.42–0.95)	0.03

**(B)**
	**ADC cases, *n* = 630**	**Controls, *n* = 775**	**Odds ratio (95% CI)**	***p* Multiple comparisons**

**HLA-A**				
01:01	178 (28.25)	192 (24.77)	1.03 (0.80–1.32)	
02:01	267 (42.38)	321 (41.42)	0.86 (0.68–1.08)	
03:01	158 (25.08)	155 (20)	1.25 (0.96–1.62)	
11:01	60 (9.52)	76 (9.81)	0.90 (0.62–1.29)	
24:02	106 (16.83)	100 (12.9)	1.31 (0.97–1.78)	
29:02	44 (6.98)	48 (6.19)	0.98 (0.64–1.51)	
31:01	36 (5.71)	33 (4.26)	1.24 (0.76–2.03)	
32:01	43 (6.83)	46 (5.94)	1.07 (0.69–1.66)	
68:01	40 (6.35)	50 (6.45)	0.91 (0.58–1.40)	
**HLA-B**				
08:01	123 (19.52)	151 (19.48)	0.87 (0.66–1.15)	
15:01	69 (10.95)	80 (10.32)	0.99 (0.70–1.40)	
18:01	42 (6.67)	50 (6.45)	0.97 (0.63–1.50)	
27:05	38 (6.03)	58 (7.48)	0.73 (0.47–1.12)	
35:01	62 (9.84)	78 (10.06)	0.90 (0.63–1.29)	
40:01	57 (9.05)	72 (9.29)	0.89 (0.61–1.29)	
44:02	106 (16.83)	108 (13.94)	1.14 (0.85–1.54)	
44:03	55 (8.73)	57 (7.35)	1.09 (0.73–1.61)	
51:01	42 (6.67)	59 (7.61)	0.76 (0.50–1.15)	
57:01	49 (7.78)	48 (6.19)	1.14 (0.75–1.74)	
**HLA-C**				
01:02	34 (5.4)	39 (5.03)	1.01 (0.63–1.64)	
02:02	46 (7.3)	60 (7.74)	0.87 (0.58–1.31)	
03:03	63 (10)	58 (7.48)	1.23 (0.84–1.80)	
03:04	81 (12.86)	102 (13.16)	0.87 (0.63–1.20)	
04:01	114 (18.10)	125 (16.13)	1.09 (0.82–1.45)	
05:01	87 (13.81)	109 (14.06)	0.90 (0.66–1.24)	
06:02	113 (17.94)	124 (16)	1.02 (0.77–1.37)	
07:01	156 (24.76)	199 (25.68)	0.84 (0.65–1.08)	
07:02	185 (29.37)	169 (21.81)	1.41 (1.09–1.81)	*0.09*
08:02	26 (4.13)	43 (5.55)	0.66 (0.40–1.10)	
12:03	58 (9.21)	49 (6.32)	1.39 (0.93–2.09)	
16:01	40 (6.35)	47 (6.06)	0.92 (0.59–1.44)	
**DRB1**				
01:01	104 (16.51)	110 (14.19)	1.18 (0.88–1.59)	
03:01	135 (21.43)	175 (22.58)	0.89 (0.69–1.16)	
04:04	66 (10.48)	65 (8.39)	1.27 (0.88–1.83)	
07:01	138 (21.9)	178 (22.97)	0.93 (0.72–1.21)	
08:01	28 (4.44)	40 (5.16)	0.81 (0.49–1.34)	
**DQB1**				
05:01	137 (21.75)	139 (17.94)	1.25 (0.96–1.64)	
06:04	20 (3.17)	51 (6.58)	0.46 (0.27–0.78)	0.02

*ADC, cervical adenocarcinoma; models adjusted for age and study*.

Of the remaining 38 alleles examined in Table 1B, we observed a statistically significant decreased association with HLA-DQB1*06:04 (OR = 0.46, 95% CI: 0.27–0.78) which retained significance after adjustment for multiple comparisons (*p* = 0.02), and an increased risk associations for HLA-C*07:02 (OR = 1.41, 95% CI: 1.09–1.81), which did not retain statistical significance after adjustments for multiple comparisons (*p* = 0.09).

We also investigated the associations that were statistically significantly associated with ADC in this analysis with risk of SCC in data from the pooled studies (*n* = 660 SCC cases), and observed that the associations for these HLA alleles, were similar between the two histologic types as shown in Table 2. Associations between all HLA alleles and SCC and ADC are presented in Table S4 in Supplementary Material.

**Table 2:** **Association between HLA alleles and cervical adenocarcinoma (ADC) and squamous cell carcinoma (SCC), among Caucasians, Pooled Western and Eastern US cervical cancer studies**.

	SCC cases odds ratio (95%CI)	ADC cases odds ratio (95%CI)	*p*_heterogeneity_
	*N* = 512	*N* = 603	
**HLA-B**			
07:02	1.29 (1.00–1.68)	1.38 (1.1–1.78)	0.62
**HLA-C**			
07:02	1.18 (0.91–1.53)	1.39 (1.1–1.79)	0.22
**DRB1**			
13:01	0.74 (0.50–1.09)	0.61 (0.40–0.91)	0.39
13:02	0.52 (0.33–0.82)	0.48 (0.30–0.76)	0.78
**DQB1**			
06:03	0.78 (0.53–1.14)	0.63 (0.42–0.94)	0.32
06:04	0.66 (0.41–1.07)	0.45 (0.26–0.76)	0.20

*p-Value for heterogeneity between SCC and ADC odds ratios from polytomous regression models; models adjusted for age and study; reference category is the pooled controls; SCC, cervical squamous cell carcinoma; ADC, cervical adenocarcinoma.

In exploratory analyses, we examined the association of HLA alleles stratified by the case HPV status restricted to the Western study as described in the Section “Materials and Methods.” Because we observed no differences in HLA allele associations by histologic subtype, we combined ADC and SCC cases for this analysis resulting in *n* = 302 HPV16 cases, *n* = 113 HPV18 cases, compared with *N* = 474 controls (Table 3). Four alleles showed increased risk with HPV18-associated cancers but decreased risk with HPV16-associated cancers (A*01:01 *p*_heterogeneity_ = 0.0003, B*08:01 *p*_heterogeneity_ = 0.003, B*15:01 *p*_heterogeneity_ = 0.003 and DRB1*03:01 *p*_heterogeneity_ = 0.01). None of these alleles was associated with ADC in the analysis of all HPV types combined (shown in Table 2). Four alleles were associated with decreased risk of HPV18-associated cancers but increased risk or no associations with HPV16-associated cancers (DRB1*07:01 *p*_heterogeneity_ = 0.001, A*02:01 *p*_heterogeneity_ = 0.006, C*05:01 *p*_heterogeneity_ = 0.01, and DRB1*04:01 *p*_heterogeneity_ = 0.01).

**Table 3:** **Association between HLA alleles and cervical cancer (regardless of histology) by HPV status, among Caucasians in the Western cervical cancer study**.

	HPV16 cases	*p*	HPV18 cases	*p*	*p*_heterogeneity_*
	*N* = 302		*N* = 113	
	OR (95% CI)		OR (95% CI)	
**HLA-A**					
01:01	0.80 (0.58–1.10)	0.17	1.82 (1.20–2.77)	0.005	0.0003
02:01	1.00 (0.75–1.34)	0.99	0.53 (0.35–0.82)	0.004	0.006
03:01	1.43 (1.03–1.97)	0.03	1.32 (0.83–2.09)	0.24	0.74
11:01	0.95 (0.60–1.50)	0.82	1.06 (0.57–1.98)	0.86	0.75
24:02	1.17 (0.78–1.74)	0.45	1.30 (0.75–2.24)	0.35	0.72
29:02	1.06 (0.63–1.80)	0.81	0.75 (0.32–1.73)	0.50	0.43
31:01	1.09 (0.58–2.02)	0.79	2.23 (1.11–4.50)	0.03	0.06
32:01	1.21 (0.70–2.12)	0.49	0.93 (0.40–2.17)	0.87	0.55
68:01	1.05 (0.60–1.84)	0.87	1.44 (0.70–2.95)	0.32	0.41
**HLA-B**					
07:02	1.29 (0.93–1.78)	0.12	1.31 (0.84–2.06)	0.24	0.94
08:01	0.61 (0.43–0.88)	0.008	1.32 (0.84–2.06)	0.23	0.003
15:01	0.49 (0.29–0.84)	0.01	1.38 (0.78–2.46)	0.27	0.003
18:01	0.97 (0.56–1.68)	0.91	1.06 (0.50–2.27)	0.88	0.82
27:05	0.63 (0.35–1.13)	0.12	0.69 (0.30–1.59)	0.38	0.83
35:01	1.02 (0.65–1.62)	0.92	1.10 (0.59–2.07)	0.76	0.83
40:01	1.11 (0.71–1.71)	0.65	0.72 (0.36–1.46)	0.36	0.25
44:02	1.93 (1.33–2.78)	0.0005	1.10 (0.63–1.94)	0.74	0.05
44:03	1.41 (0.89–2.22)	0.15	0.62 (0.27–1.43)	0.26	0.06
51:01	1.05 (0.65–1.71)	0.83	0.52 (0.21–1.24)	0.14	0.12
57:01	1.44 (0.88–2.36)	0.15	0.77 (0.34–1.78)	0.54	0.15
**HLA-C**					
01:02	0.79 (0.42–1.50)	0.47	1.47 (0.69–3.10)	0.32	0.15
02:02	0.91 (0.55–1.51)	0.70	0.64 (0.28–1.45)	0.28	0.42
03:03	0.70 (0.40–1.22)	0.21	1.40 (0.74–2.66)	0.31	0.06
03:04	0.84 (0.56–1.26)	0.40	0.81 (0.45–1.45)	0.49	0.91
04:01	1.01 (0.69–1.47)	0.96	1.00 (0.58–1.71)	0.995	0.97
05:01	1.45 (0.995–2.12)	0.053	0.61 (0.31–1.19)	0.14	0.01
06:02	1.17 (0.82–1.67)	0.38	0.69 (0.39–1.24)	0.21	0.08
07:01	0.74 (0.54–1.03)	0.07	1.08 (0.69–1.67)	0.74	0.12
07:02	1.28 (0.94–1.77)	0.12	1.29 (0.82–2.01)	0.27	0.99
08:02	0.49 (0.24–1.01)	0.054	0.66 (0.25–1.73)	0.40	0.59
12:03	0.89 (0.50–1.58)	0.69	1.78 (0.92–3.43)	0.09	0.06
16:01	1.30 (0.78–2.16)	0.31	0.66 (0.27–1.61)	0.36	0.14
**DRB1**					
01:01	1.09 (0.74–1.59)	0.67	1.45 (0.87–2.42)	0.15	0.29
03:01	0.61 (0.43–0.86)	0.005	1.12 (0.71–1.75)	0.63	0.02
04:01	1.54 (1.11–2.14)	0.01	0.74 (0.42–1.30)	0.29	0.01
04:04	0.64 (0.37–1.10)	0.11	0.94 (0.46–1.92)	0.86	0.34
07:01	1.41 (1.03–1.92)	0.03	0.56 (0.32–0.96)	0.04	0.001
08:01	0.91 (0.50–1.67)	0.76	1.50 (0.71–3.16)	0.29	0.23
11:01	1.89 (1.13–3.15)	0.02	2.08 (1.05–4.13)	0.04	0.78
13:01	0.62 (0.37–1.03)	0.06	0.45 (0.19–1.08)	0.07	0.51
13:02	0.66 (0.38–1.14)	0.14	0.37 (0.13–1.04)	0.06	0.29
15:01	0.97 (0.71–1.33)	0.87	1.47 (0.96–2.26)	0.08	0.07
**DQB1**					
03:01	1.33 (0.99–1.78)	0.06	1.07 (0.69–1.66)	0.77	0.36
03:02	0.91 (0.65–1.28)	0.60	0.90 (0.55–1.47)	0.66	0.94
03:03	1.59 (1.04–2.43)	0.03	0.99 (0.50–1.96)	0.97	0.18
05:01	1.05 (0.74–1.50)	0.77	1.42 (0.88–2.29)	0.15	0.24
06:02	0.89 (0.65–1.22)	0.47	1.41 (0.92–2.17)	0.12	0.05
06:03	0.64 (0.39–1.05)	0.07	0.51 (0.22–1.14)	0.10	0.60
06:04	0.75 (0.41–1.37)	0.35	0.24 (0.06–1.03)	0.06	0.14

*p-Value for heterogeneity between HPV16 and HPV18 odds ratios from polytomous regression models. Polytomous regression used to estimate odds ratios (OR) between HLA alleles and tumors containing HPV16 vs. HPV18 DNA; reference category are controls from the Western cervical cancer study. Models adjusted for age.

Table 4 shows the results of the unphased allele combinations that are in high linkage disequilibrium and found to be associated with risk of ADC. We observed a statistically significant elevated risk of ADC associated with carriers of HLA-B*07:02 and HLA-C*07:02 (OR = 1.33, 95% CI: 1.01–1.76). We also observed a statistically significant decreased risk of ADC associated with HLA-DRB1*13:02 and DQB1*06:04 (OR = 0.41, 95% CI: 0.21–0.80), and suggestive decreased risk for HLA-DRB1*13:01 and DQB1*06:03 (OR = 0.62, 95% CI: 0.38–1.03). Evaluating these combinations among SCC cases vs. controls revealed similar patterns of association (data not shown).

**Table 4:** **Association between HLA allele combinations and cervical adenocarcinoma among Caucasians and pooled Western and Eastern US cervical cancer studies**.

B*07:02	C*07:02	DRB1*13:01	DRB1*13:02	DQB1*06:03	DQB1*06:04	ADC cases (*n* = 574)	Controls (*n* = 656)	OR (95% CI)
–	–	–	–	–	–	335 (58.36)	365 (55.64)	1.0 (Ref)
–	C*07:02	–	–	–	–	10 (1.74)	12 (1.83)	1.03 (0.44–2.45)
–	–	–	DRB1*13:02	–	–	7 (1.22)	14 (2.13)	0.54 (0.21–1.37)
B*07:02	C*07:02	–	–	–	–	156 (27.18)	132 (20.12)	1.33 (1.01–1.76)
–	–	DRB1*13:01	–	DQB1*06:03	–	28 (4.88)	55 (8.38)	0.62 (0.38–1.03)
–	–	–	DRB1*13:02	–	DQB1*06:04	13 (2.26)	36 (5.49)	0.41 (0.21–0.80)
B*07:02	C*07:02	DRB1*13:01	–	DQB1*06:03	–	8 (1.39)	9 (1.37)	1.07 (0.40–2.88)
B*07:02	C*07:02	–	DRB1*13:02	–	DQB1*06:03	7 (1.22)	6 (0.91)	1.27 (0.42–3.87)

*Model adjusted for age and study. Only combinations of over 1% in either cases or controls are shown in this table; “–” indicates absence of the respective allele(s)*.

## Discussion

Cervical ADC is less common than cervical SCC, comprising between 5 to 20% of histologic subtype of cervical cancer depending on region (2), however, age-adjusted rates of ADC have increased by ~32% over the past 35 years in the US, while they have declined for SCC (26). This combined study represents the largest study to examine the risk of ADC associated with HLA region loci. Results from this pooled analysis of 630 ADC cases and 775 controls affirmed findings from prior studies of cervical cancer that largely represented SCC. We observed an increased ADC risk associated with selected *a priori* alleles at B*07:02 and decreased ADC risk with DRB1*13:01, DRB1*13:02, and DQB1*06:03. Among new findings of this study, we identified a decreased risk of ADC with HLA class II allele DQB1*06:04, which remained significant after correction for multiple comparison. This allele has not been reported in other studies that examined HLA associations with cervical SCC or without regard to histologic type. The prevalence of DQB1*06:04 was 7.2% in the combined control population (6.6% in the Western and 8.8% in the Eastern study controls, *p* > 0.05), and it was significantly correlated with DRB1*13:02 (correlation coefficient of 0.83, *p* < 0.0001), suggesting that the inverse association we observed is likely due to linkage disequilibrium or its combination with DRB1*13:02, an allele that has been reported to be inversely associated with cervical cancer in other studies (20, 27).

Our data also strongly suggest that the associations we observed between HLA alleles and ADC do not differ substantially from those reported for SCC. The previously reported decreased association between SCC and HLA class II DRB1*13:01 was shown in this study to also be associated with ADC (14, 15, 19, 27, 28). We also showed the increased risk of class I HLA-B*07:02 with ADC as shown with SCC (14, 27). Our observation that HLA associations were not different by histology indicates that with cervical cancer, HLA presentation of foreign peptides is independent of histology. Further pooling of HLA results with other studies may enable a more robust analysis of HLA types that recognize HPV type-specific peptides, as our exploratory analysis of HPV16 vs. HPV18 cervical cancer suggests (Table 3).

While the HPV type-specific analyses presented are based on small sample size, it raises the possibility that specific HLA alleles could be mechanistically important in clearing or tolerance of HPV infections. We note that our sample size was limited, thus our findings could be due to false-positivity, and replication in other settings is warranted.

We observed that increased risk of ADC was associated with the combination of HLA B*07:02 and C*07:02, which is present in over 15% of cases and controls. In addition, the correlation coefficient for the two was 0.99, indicating that they are in high linkage disequilibrium. We cannot determine if the increased risk is associated more with one of these markers or if they together may indicate risk of an untagged marker. Similarly, DRB1*13:02 and DQB1*06:04 were very rare (under 2%) by themselves, while in combination, they occurred in over 2% of the cases and 5% of the controls. This observation likewise indicates that the inverse association may be due to the individual alleles, the combination of alleles, or some marker in linkage with the combination of alleles.

The large sample size, well-characterized population-based studies, and high-resolution molecular typing of HLA alleles are substantial strengths of this analysis. There are several limitations to be considered. The HLA genotyping was performed in two different regions of the country, and genetic background may be different. Even though we restricted to Caucasian ancestry and controlled for study in the models, we may not have eliminated residual confounding from population stratification. Furthermore, it is possible that general laboratory differences in practice and protocol could have led to the non-differential misclassification of the exposure and thus the attenuation of associations. Since both studies were restricted to those with Caucasian ancestry, there may be different allele frequencies within different geographic regions or subpopulations of the US. Even so, none of the alleles identified as being statistically significantly associated with ADC differed by study.

Another limitation was that, for the Western study, not all biopsies or surgical tissues were available for HPV DNA typing of tumor tissue. While we can assume most if not all cervical cancer cases were HPV-positive for one or more carcinogenic types (29–31), our HPV16/18 analyses are thus limited and need replication.

In summary, pooling of these two studies provided a large sample size to assess the role of HLA on the risk of ADC, and provided evidence that the HLA class I and II associations for ADC are similar to those reported for SCC. Larger studies with detailed HPV assessment coupled with high quality genetic data are needed to further evaluate the effects of HLA and cervical ADC by HPV genotype.

## Conflict of Interest Statement

The authors declare that the research was conducted in the absence of any commercial or financial relationships that could be construed as a potential conflict of interest.

## Supplementary Material

The Supplementary Material for this article can be found online at http://www.frontiersin.org/Journal/10.3389/fonc.2014.00119/abstract
