# Supplementary material for: Human Leukocyte Antigen Class I and II Alleles and Cervical Adenocarcinoma
Source: Front Oncol. 2014 Jun 19;4:119. doi: 10.3389/fonc.2014.00119 (PMC4062965; doi:10.3389/fonc.2014.00119)
Supplement: Supplementary file 1 [file DataSheet_1.DOCX]

Supplementary Table 1: Previous studies of HLA alleles and cervical cancer risk

| Allele | Reference (PMIDs listed) |
| --- | --- |
| B*07:02 | 22320938 (32), 11679920 (19), 9829713 (15), 18351579 (33), 23482656 (14) |
|  |  |
| DRB1 |  |
| 04:01 | 19272325 (34), 10755413 (35) |
| 11:01 | 11588129 (36), 12447731 (12) |
| 13:01 | 8682580 (37) , 9829713 (15), 11679920 (19), 12447731 (12), 16720214 (27), 17538887 (38), 19585495 (28) |
| 13:02 | 8682580 (37), 9829713 (15), 12447731 (12), 16720214 (27), 17538887 (38) |
| 15:01 | 9829713 (15), 10755413 (35), 11519043 (39), 12691704 (40), 16720214 (27), 18351579 (33), 18451182 (20), 23482656 (14) |
|  |  |
| DQB1 |  |
| 03:01 | 8529095 (41), 8782644 (42), 9805655 (43), 11588129 (36), 12447731 (12), 17379283 (44), 18650831 (45) |
| 03:02 | 9829713 (15), 11679920 (19), 17379283 (44), 18820539 (46) |
| 03:03 | 10636516 (47), 17379283 (44) |
| 06:02 | 9533756 (48), 9829713 (15), 11519043 (39), 17379283 (44), 18351579 (33), 18650831 (45) |
| 06:03 | 9805655 (43), 17379283 (44), 18650831 (45) |

Supplementary Table 2: Distribution of selected characteristics among cervical adenocarcinoma cases and controls

| Characteristics |  | Western US Adenocarcinoma Study | |  | Eastern US Adenocarcinoma Study | |  | Pooled Eastern & Western Studies | |
| --- | --- | --- | --- | --- | --- | --- | --- | --- | --- |
|  |  | Cases  n=468 | Controls n=513 |  | Cases  n=162 | Controls n=262 |  | Cases n=630 | Controls n=775 |
| Age (median) | <40 | 279 (59.62) | 236 (46.0) |  | 73 (57.94) | 145 (55.34) |  | 352 (59.26) | 381 (49.16) |
|  | 40+ | 189 (40.38) | 277 (54.0) |  | 53 (42.06) | 117 (44.66) |  | 242 (40.74) | 394 (50.84) |
|  |  |  |  |  |  |  |  |  |  |
| Smoking status | Never | 252 (53.85) | 249 (48.54) |  | 65 (51.59) | 137 (52.29) |  | 317 (53.37) | 386 (49.81) |
|  | Former | 119 (25.43) | 153 (29.82) |  | 40 (31.75) | 69 (26.34) |  | 159 (26.77) | 222 (28.65) |
|  | Current | 97 (20.73) | 111 (21.64) |  | 21 (16.67) | 56 (21.37) |  | 118 (19.87) | 167 (21.55) |
|  |  |  |  |  |  |  |  |  |  |
| Lifetime partner | 0-1 | 57 (12.18) | 124 (24.17) |  | 25 (20.16) | 91 (34.87) |  | 82 (13.85) | 215 (27.78) |
|  | 2+ | 411 (87.82) | 389 (75.83) |  | 99 (79.84) | 170 (65.13) |  | 510 (86.15) | 559 (72.22) |
|  |  |  |  |  |  |  |  |  |  |
| HPV DNA status* | HPV- | 32 (11.59^∏^) | *** |  | 15 (35.71^∏^) | *** |  | 47 (14.78^∏^) |  |
|  | HPV16+ | 117 (42.39) |  |  | 12 (28.57) |  |  | 129 (40.57) |  |
|  | HPV18+ | 91 (32.97) |  |  | 10 (23.81) |  |  | 101 (31.70) |  |
|  | HPV16+ & 18+ | 26 (9.42) |  |  | 1 (2.38) |  |  | 27 (8.49) |  |
|  | Other | 10 (3.62) |  |  | 4 (9.52) |  |  | 14 (4.40) |  |
|  | Missing/HPV results post-treatment | 192 |  |  | 120** |  |  | 312 |  |
|  |  |  |  |  |  |  |  |  |  |

*Tumor samples were evaluated in the Western US Adenocarcinoma study; exfoliated cervical cells were evaluated in the Eastern US Adenocarcinoma study

^∏^Percentages calculated among those with available HPV results (thus excludes missing those with missing HPV results from the denominator)

**There were 44 records with missing HPV results, and 77 where HPV results were post-treatment. This occurred only in the Eastern Study

***Control HPV data are not presented as these are only available from the Eastern study. Since it is not used in the analysis of the combined data, we do not present in the table.

Supplementary Table 3: Alleles with genotype frequencies over 5% among controls from each study and in the pooled sample

|  | Western US |  | Eastern US |  |  |  | Pooled Eastern and Western |
| --- | --- | --- | --- | --- | --- | --- | --- |
|  | n (%) |  | n (%) |  | p-value* |  | n=775 (%: based on 648) |
| HLA-A |  |  |  |  |  |  |  |
| 01:01 | 148 (31.22) |  | 44 (25.29) |  |  |  | 192 (29.63) |
| 02:01 | 243 (51.27) |  | 78 (44.83) |  |  |  | 321 (49.54) |
| 03:01 | 115 (24.26) |  | 40 (22.99) |  |  |  | 155 (23.92) |
| 11:01 | 57 (12.03) |  | 19 (10.92) |  |  |  | 76 (11.73) |
| 24:02 | 68 (14.35) |  | 32 (18.39) |  |  |  | 100 (15.43) |
| 29:02 | 37 (7.81) |  | 11 (6.32) |  |  |  | 48 (7.41) |
| 31:01 | 26 (5.49) |  | 7 (4.02) |  |  |  | 33 (5.09) |
| 32:01 | 31 (6.54) |  | 15 (8.62) |  |  |  | 46 (7.10) |
| 68:01 | 33 (6.96) |  | 17 (9.77) |  |  |  | 50 (7.72) |
| HLA-B |  |  |  |  |  |  |  |
| 07:02 | 123 (25.95) |  | 39 (21.91) |  |  |  | 162 (24.85) |
| 08:01 | **121 (25.53)** |  | **30 (16.85)** |  | **0.02** |  | 151 (23.16) |
| 15:01 | 57 (12.03) |  | 23 (12.92) |  |  |  | 80 (12.27) |
| 18:01 | 36 (7.59) |  | 14 (7.87) |  |  |  | 50 (7.67) |
| 27:05 | 41 (8.65) |  | 17 (9.55) |  |  |  | 58 (8.90) |
| 35:01 | 53 (11.18) |  | 25 (14.04) |  |  |  | 78 (11.96) |
| 40:01 | 57 (12.03) |  | 15 (8.43) |  |  |  | 72 (11.04) |
| 44:02 | **69 (14.56)** |  | **39 (21.91)** |  | **0.02** |  | 108 (16.56) |
| 44:03 | 45 (9.49) |  | 12 (6.74) |  |  |  | 57 (8.74) |
| 51:01 | 46 (9.70) |  | 13 (7.30) |  |  |  | 59 (9.05) |
| 57:01 | 38 (8.02) |  | 10 (5.62) |  |  |  | 48 (7.36) |
| HLA-C |  |  |  |  |  |  |  |
| 01:02 | 30 (6.32) |  | 9 (5.11) |  |  |  | 39 (5.99) |
| 02:02 | 45 (9.47) |  | 15 (8.52) |  |  |  | 60 (9.22) |
| 03:03 | 43 (9.05) |  | 15 (8.52) |  |  |  | 58 (8.91) |
| 03:04 | 81 (17.05) |  | 21 (11.93) |  |  |  | 102 (15.67) |
| 04:01 | 83 (17.47) |  | 42 (23.86) |  |  |  | 125 (19.20) |
| 05:01 | **71 (14.95)** |  | **38 (21.59)** |  | **0.04** |  | 109 (16.74) |
| 06:02 | 90 (18.95) |  | 34 (19.32) |  |  |  | 124 (19.05) |
| 07:01 | 147 (30.95) |  | 52 (29.55) |  |  |  | 199 (30.57) |
| 07:02 | 129 (27.16) |  | 40 (22.73) |  |  |  | 169 (25.96) |
| 08:02 | 32 (6.74) |  | 11 (6.25) |  |  |  | 43 (6.61) |
| 12:03 | 35 (7.37) |  | 14 (7.95) |  |  |  | 49 (7.53) |
| 16:01 | 37 (7.79) |  | 10 (5.68) |  |  |  | 47 (7.22) |
| DRB1 |  |  |  |  |  |  |  |
| 01:01 | 78 (15.23) |  | 32 (16.58) |  |  |  | 110 (15.60) |
| 03:01 | 135 (26.37) |  | 40 (20.73) |  |  |  | 175 (24.82) |
| 04:01 | 98 (19.14) |  | 33 (17.10) |  |  |  | 131 (18.58) |
| 04:04 | 48 (9.38) |  | 17 (8.81) |  |  |  | 65 (9.22) |
| 07:01 | 131 (25.59) |  | 47 (24.35) |  |  |  | 178 (25.25) |
| 08:01 | 30 (5.86) |  | 10 (5.18) |  |  |  | 40 (5.67) |
| 11:01 | **30 (5.86)** |  | **27 (13.99)** |  | **0.0004** |  | 57 (8.09) |
| 13:01 | 55 (10.74) |  | 18 (9.33) |  |  |  | 73 (10.35) |
| 13:02 | 45 (8.79) |  | 21 (10.88) |  |  |  | 66 (9.36) |
| 15:01 | **140 (27.34)** |  | **33 (17.10)** |  | **0.005** |  | 173 (24.54) |
| DQB1 |  |  |  |  |  |  |  |
| 03:01 | **150 (29.30)** |  | **76 (39.38)** |  | **0.01** |  | 226 (32.06) |
| 03:02 | 117 (22.85) |  | 41 (21.24) |  |  |  | 158 (22.41) |
| 03:03 | 50 (9.77) |  | 15 (7.77) |  |  |  | 65 (9.22) |
| 05:01 | 97 (18.95) |  | 42 (21.76) |  |  |  | 139 (19.72) |
| 06:02 | **140 (27.34)** |  | **32 (16.58)** |  | **0.003** |  | 172 (24.40) |
| 06:03 | 58 (11.33) |  | 17 (8.81) |  |  |  | 75 (10.64) |
| 06:04 | 34 (6.64) |  | 17 (8.81) |  |  |  | 51 (7.23) |

*p-value for differences in the frequencies of the two studies.

| Supplementary Table 4: Association between all HLA alleles and cervical adenocarcinoma (ADC) and squamous cell carcinoma (SCC), among Caucasians, Pooled Western and Eastern U.S. Cervical Cancer Studies   \|  \| **SCC**  **N=512** \| **ADC**  **N=603** \|  \| \| --- \| --- \| --- \| --- \| \|  \| OR (95% CI) \| OR (95% CI) \| p_het*_ \| \| **HLA-A** \|  \|  \|  \| \| 01:01 \| 1.08 (0.84-1.39) \| 1.03 (0.81-1.32) \| 0.74 \| \| 02:01 \| 0.86 (0.68-1.08) \| 0.86 (0.69-1.09) \| 0.93 \| \| 03:01 \| 1.39 (1.07-1.81) \| 1.25 (0.96-1.62) \| 0.43 \| \| 11:01 \| 1.02 (0.71-1.46) \| 0.89 (0.62-1.28) \| 0.49 \| \| 24:02 \| 1.10 (0.80-1.51) \| 1.33 (0.98-1.80) \| 0.25 \| \| 29:02 \| 0.76 (0.47-1.22) \| 0.99 (0.65-1.53) \| 0.27 \| \| 31:01 \| 0.93 (0.54-1.58) \| 1.23 (0.75-2.01) \| 0.29 \| \| 32:01 \| 1.25 (0.82-1.93) \| 1.06 (0.68-1.64) \| 0.45 \| \| 68:01 \| 1.00 (0.65-1.55) \| 0.91 (0.59-1.40) \| 0.66 \| \| **HLA-B** \|  \|  \|  \| \| 07:02 \| 1.29 (1.00-1.68) \| 1.38 (1.07-1.78) \| 0.62 \| \| 08:01 \| 0.84 (0.64-1.12) \| 0.88 (0.67-1.15) \| 0.81 \| \| 15:01 \| 0.52 (0.35-0.80) \| 0.99 (0.70-1.41) \| 0.004 \| \| 18:01 \| 0.89 (0.57-1.40) \| 0.98 (0.64-1.51) \| 0.67 \| \| 27:05 \| 0.58 (0.37-0.93) \| 0.71 (0.46-1.10) \| 0.43 \| \| 35:01 \| 0.75 (0.51-1.10) \| 0.89 (0.62-1.27) \| 0.40 \| \| 40:01 \| 1.22 (0.86-1.74) \| 0.90 (0.62-1.31) \| 0.12 \| \| 44:02 \| 1.51 (1.13-2.03) \| 1.12 (0.83-1.50) \| 0.04 \| \| 44:03 \| 1.14 (0.77-1.70) \| 1.09 (0.73-1.61) \| 0.81 \| \| 51:01 \| 1.11 (0.75-1.65) \| 0.77 (0.51-1.16) \| 0.09 \| \| 57:01 \| 1.11 (0.72-1.70) \| 1.15 (0.76-1.76) \| 0.85 \| \| **HLA-C** \|  \|  \|  \| \| 01:02 \| 1.09 (0.68-1.76) \| 1.03 (0.64-1.66) \| 0.81 \| \| 02:02 \| 0.71 (0.46-1.10) \| 0.88 (0.59-1.33) \| 0.35 \| \| 03:03 \| 1.03 (0.69-1.55) \| 0.23 (0.84-1.79) \| 0.40 \| \| 03:04 \| 0.92 (0.67-1.28) \| 0.89 (0.65-1.22) \| 0.82 \| \| 04:01 \| 0.80 (0.59-1.09) \| 1.07 (0.81-1.43) \| 0.07 \| \| 05:01 \| 1.29 (0.96-1.74) \| 0.87 (0.64-1.19) \| 0.01 \| \| 06:02 \| 1.01 (0.75-1.36) \| 1.02 (0.76-1.35) \| 0.96 \| \| 07:01 \| 0.89 (0.69-1.14) \| 0.84 (0.65-1.08) \| 0.70 \| \| 07:02 \| 1.18 (0.91-1.53) \| 1.39 (1.08-1.79) \| 0.22 \| \| 08:02 \| 0.92 (0.57-1.48) \| 0.68 (0.41-1.13) \| 0.27 \| \| 12:03 \| 1.01 (0.65-1.57) \| 1.42 (0.95-2.13) \| 0.12 \| \| 16:01 \| 0.83 (0.52-1.32) \| 0.93 (0.60-1.45) \| 0.62 \| \| **DRB1** \|  \|  \|  \| \| 01:01 \| 1.03 (0.76-1.40) \| 1.18 (0.88-1.59) \| 0.39 \| \| 03:01 \| 0.74 (0.56-0.97) \| 0.89 (0.69-1.16) \| 0.19 \| \| 04:01 \| 1.31 (1.00-1.72) \| 1.10 (0.83-1.46) \| 0.23 \| \| 04:04 \| 0.78 (0.52-1.18) \| 1.29 (0.89-1.85) \| 0.02 \| \| 07:01 \| 1.21 (0.87-1.69) \| 2.30 (1.70-3.11) \| 0.00005 \| \| 08:01 \| 0.71 (0.42-1.19) \| 0.80 (0.49-1.33) \| 0.65 \| \| 11:01 \| 1.73 (1.19-2.51) \| 1.28 (0.86-1.90) \| 0.12 \| \| 13:01 \| 0.74 (0.50-1.09) \| 0.61 (0.40-0.91) \| 0.39 \| \| 13:02 \| 0.52 (0.33-0.82) \| 0.48 (0.30-0.76) \| 0.78 \| \| 15:01 \| 1.22 (0.95-1.56) \| 1.20 (0.94-1.55) \| 0.92 \| \| **DQB1** \|  \|  \|  \| \| 03:01 \| 1.46 (1.16-1.84) \| 1.14 (0.90-1.45) \| 0.046 \| \| 03:02 \| 0.83 (0.63-1.09) \| 1.16 (0.90-1.51) \| 0.02 \| \| 03:03 \| 1.27 (0.89-1.83) \| 1.06 (0.73-1.55) \| 0.35 \| \| 05:01 \| 1.01 (0.77-1.34) \| 1.25 (0.95-1.63) \| 0.15 \| \| 06:02 \| 1.16 (0.90-1.49) \| 1.15 (0.89-1.48) \| 0.96 \| \| 06:03 \| 0.78 (0.53-1.14) \| 0.63 (0.42-0.94) \| 0.32 \| \| 06:04 \| 0.66 (0.41-1.07) \| 0.45 (0.26-0.76) \| 0.20 \| |  |
| --- | --- | --- | --- | --- | --- | --- | --- | --- | --- | --- | --- | --- | --- | --- | --- | --- | --- | --- | --- | --- | --- | --- | --- | --- | --- | --- | --- | --- | --- | --- | --- | --- | --- | --- | --- | --- | --- | --- | --- | --- | --- | --- | --- | --- | --- | --- | --- | --- | --- | --- | --- | --- | --- | --- | --- | --- | --- | --- | --- | --- | --- | --- | --- | --- | --- | --- | --- | --- | --- | --- | --- | --- | --- | --- | --- | --- | --- | --- | --- | --- | --- | --- | --- | --- | --- | --- | --- | --- | --- | --- | --- | --- | --- | --- | --- | --- | --- | --- | --- | --- | --- | --- | --- | --- | --- | --- | --- | --- | --- | --- | --- | --- | --- | --- | --- | --- | --- | --- | --- | --- | --- | --- | --- | --- | --- | --- | --- | --- | --- | --- | --- | --- | --- | --- | --- | --- | --- | --- | --- | --- | --- | --- | --- | --- | --- | --- | --- | --- | --- | --- | --- | --- | --- | --- | --- | --- | --- | --- | --- | --- | --- | --- | --- | --- | --- | --- | --- | --- | --- | --- | --- | --- | --- | --- | --- | --- | --- | --- | --- | --- | --- | --- | --- | --- | --- | --- | --- | --- | --- | --- | --- | --- | --- | --- | --- | --- | --- | --- | --- | --- | --- | --- | --- | --- | --- | --- | --- | --- | --- | --- | --- | --- | --- | --- | --- | --- | --- | --- | --- | --- | --- | --- | --- | --- | --- |

*p_het=_p-value for heterogeneity between SCC and ADC.

Polytomous regression used to estimate odds ratios (OR) between HLA alleles and cervical squamous cell carcinoma (SCC) and adenocarcinomas (ADC). Models adjusted for age and study.

Supplementary Figure 1: Consort Diagram describing the pooled Western and Eastern US cervical Adenocarcinoma studies
